# Dietary Pseudopurpurin Improves Bone Geometry Architecture and Metabolism in Red-Bone Guishan Goats

**DOI:** 10.1371/journal.pone.0037469

**Published:** 2012-05-18

**Authors:** ChenChen Wu, XiaoBing Li, TieSuo Han, Peng Li, JianGuo Wang, GuoWen Liu, Zhe Wang, ChangRong Ge, ShiZheng Gao

**Affiliations:** 1 College of Animal Science and Veterinary Medicine, Jilin University, Jilin, People's Republic of China; 2 College of Animal Science and Veterinary Medicine, Shenyang Agriculture University, Liaoning, People's Republic of China; 3 College of Animal Food and Science Technology, Yunnan Agriculture University, Yunnan, People's Republic of China; Illinois Institute of Technology, United States of America

## Abstract

Red-colored bones were found initially in some Guishan goats in the 1980s, and they were designated red-boned goats. However, it is not understood what causes the red color in the bone, or whether the red material changes the bone geometry, architecture, and metabolism of red-boned goats. Pseudopurpurin was identified in the red-colored material of the bone in red-boned goats by high-performance liquid chromatography–electrospray ionization–mass spetrometry and nuclear magnetic resonance analysis. Pseudopurpurin is one of the main constituents of *Rubia cordifolia L*, which is eaten by the goats. The assessment of the mechanical properties and micro-computed tomography showed that the red-boned goats displayed an increase in the trabecular volume fraction, trabecular thickness, and the number of trabeculae in the distal femur. The mean thickness, inner perimeter, outer perimeter, and area of the femoral diaphysis were also increased. In addition, the trabecular separation and structure model index of the distal femur were decreased, but the bone mineral density of the whole femur and the mechanical properties of the femoral diaphysis were enhanced in the red-boned goats. Meanwhile, expression of alkaline phosphatase and osteocalcin mRNA was higher, and the ratio of the receptor activator of the nuclear factor kappa B ligand to osteoprotegerin was markedly lower in the bone marrow of the red-boned goats compared with common goats. To confirm further the effect of pseudopurpurin on bone geometry, architecture, and metabolism, Wistar rats were fed diets to which pseudopurpurin was added for 5 months. Similar changes were observed in the femurs of the treated rats. The above results demonstrate that pseudopurpurin has a close affinity with the mineral salts of bone, and consequently a high level of mineral salts in the bone cause an improvement in bone strength and an enhancement in the structure and metabolic functions of the bone.

## Introduction

Guishan goats are a rare, wild native breed of ***Capra hircus*** present in China; they are categorized among the “First Class National Protection Animals of China”. Goats are distributed mainly in the Guishan Mountains in Yunnan province in the southwest of China. The goats have been developed through more than 20 generations of inbreeding in Yunnan province, and are maintained mainly by grazing. Red-colored bones were found initially in some Guishan goats in the 1980s, and these goats were designated “red-boned” goats to distinguish them from common goats.

The red-boned goats and the common goats that do not have red bones are in fact variants of the same species. They live in the same location and graze in the Guishan Mountains. The red-boned goats are characterized by possession of whole red-colored bones. However, the cause of the red color in the bone is unknown, and it is also unknown whether the red color in bone is caused by endogenous or exogenous factors. It is not understood whether the bone geometry, architecture and metabolism in the red-boned goats are different from those of common goats. Therefore, to increase understanding of the red-boned goats, the red material of the red-colored bone was extracted and analyzed by high-performance liquid chromatography–electrospray ionization–mass spetrometry (HPLC-ESI-MS) and nuclear magnetic resonance (NMR) spectroscopy. Moreover, bone samples from the red-boned goats were analyzed by micro-computed tomography scanning, assessment of their mechanical properties, real-time polymerase chain reaction, dual energy X-ray absorptiometry, and inductively coupled plasma mass spectrometry. To confirm further the effect of the red material on the stained bone and the bone geometry, architecture and metabolism, an experiment was conducted on Wistar rats that involved feeding diets to which the red material was added for 5 months.

## Results

### Characteristics of red-boned Guishan goats

**Figure 1:**
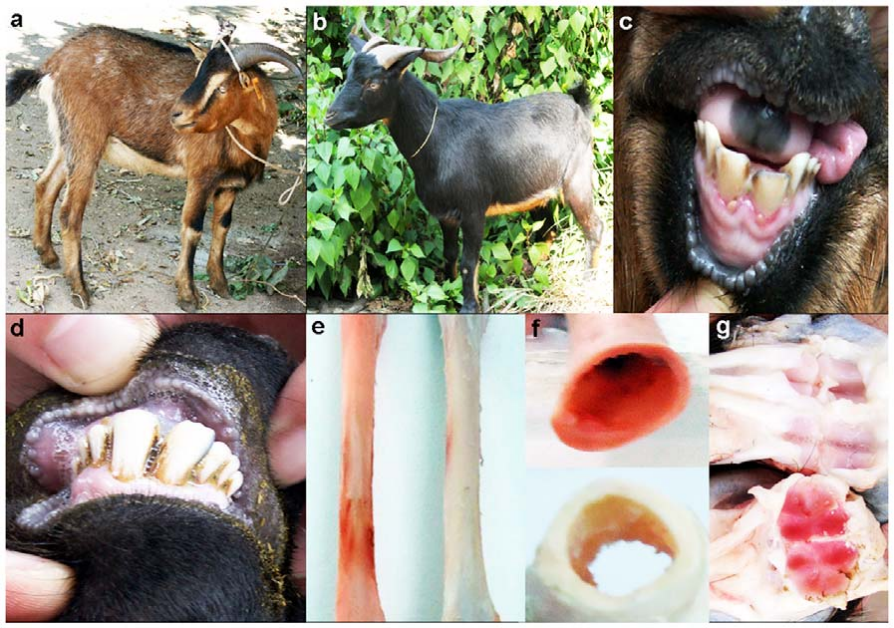
Red-boned and common goats. **a.** Red-boned goat. **b.** Common goat. **c.** The teeth of a red-boned goat. **d.** The teeth of a common goat. **e.** The femur of a red-boned goat (left) compared with that of a common goat (right) at 18 months of age. **f.** Cross-section of the femoral diaphysis from a common goat (below); the cross-section of the femoral diaphysis is red in the red-boned goat (above). **g.** The cancellous bone from a common goat (above); the whole cancellous bone is red in the red-boned goat (below) at 18 months of age.

Guishan goats are divided into red-boned goats (**Figure 1a**) and common goats (**Figure 1b**). The red-boned goats are also characterized by pink teeth (**Figure 1c**), unlike the common goats (**Figure 1d**). All of the bones in red-boned goats are red colored, especially the diaphyses of the four limb bones, the cranial bones, the scapular bone, the articular cartilage, the vertebrae and the ribs. Certain bones in red-boned goats are stained red on their periosteal and endosteal surfaces (**Figure 1e, 1f**). The whole of the cancellous bone is red in the red-boned goats, in comparison to that of the common goats (**Figure 1g**). Furthermore, we observed that the red color does not disappear from any part of the bone but becomes bright red during long exposure to light and air; it therefore seems to be light-fast.

### Analysis of the chemical structure of the red material

**Figure 2:**
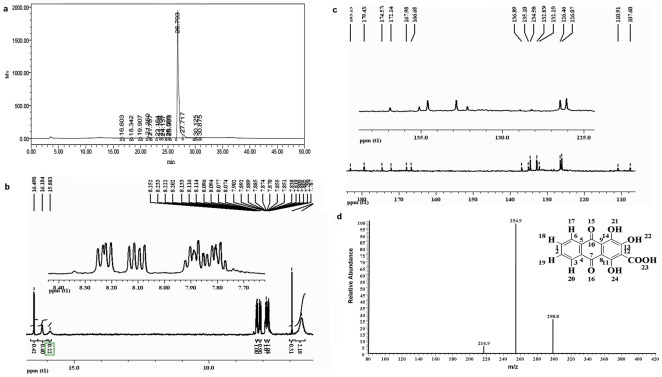
The chemical structure analysis of compound pseudopurpurin. **a.** The HPLC absorption scanning profiles of red matter from red bone tissue extracted using hydrochloric acid and methylbenzene. **b.** ^1^H NMR spectrum (400 MHz) of compound pseudopurpurin. **c.** ^13^C NMR spectrum (400 MHz) of compound pseudopurpurin. **d.** ESI-MS product ion mass spectrum and the chemical structures of compound pseudopurpurin in negative ions.

Use of HPLC revealed the presence of peaks representing the red material from the red-boned goats (**Figure 2a**). However, chromatograms of the carmine material showed only one main peak, at 26.793 min.

The stopped-flow ^1^H NMR spectrum (400 MHz, DMSO-***d_6_***) of the carmine matter showed four aromatic protons at δ 8.22 ppm (H 20) (dd, ***J*** = 8 Hz, 1 H), δ 8.10 ppm (H 17) (dd, ***J*** = 8 Hz, 1 H) and δ 7.84 ppm (H 18, 19) (m, 2 H). The ^1^H NMR spectrum was very complex, and resonances in this region could be attributed to hydroxyl and carboxyl protons of purified carmine matter as singlets at δ 7.82, δ 15.88, δ 16.18 and δ 16.49 ppm, while at δ 7.79 ppm it was disturbed by the residual NH_4_^+^ signal (**Figure 2b**). The ^13^C NMR spectrum provided fifteen signals as follows: δ 183.13 ppm (C 7), δ 179.43 ppm (C 10) and δ 174.58 (C 23) ppm for two carbonyl groups and one carboxy group, respectively; δ 172.15 ppm (C 11), δ 167.99 ppm (C 13) and δ 166.68 ppm (C 14) for hydroxy protons (C-OH); δ 134.58 ppm (C 1), δ 132.89 ppm (C 2), δ 126.46 ppm (C 6), δ 126.07 ppm (C 3), δ 110.91 ppm (C 9), δ 136.89 ppm (C 5), δ 135.10 ppm (C 4), δ 132.19 ppm (C 8) and δ 107.60 ppm (C 12) for condensed rings (ring CHs and quaternary carbons), and C-4, 5 and C-8, 9 double bonds. δ 107.60 ppm, which correlated with a carboxy group (**Figure 2c**). The parameters were as follows: UV/vis: λ_max_ 255 nm; HRMS (***m/z***): [M-H]^−^ calculated for C_15_H_8_O_7_ (pseudopurpurin), 298.8; the [M-H-CO_2_]^−^ fragment ion was found at ***m/z*** 254.9 (**Figure 2d**).

### Bone mineral elements

In the distal femur and the femur diaphysis, there were no significant differences in sodium (Na), sulfur (S) and potassium (K) between red-boned and common goats (***P***>0.05); the levels of calcium (Ca), phosphorus (P), magnesium (Mg), zinc (Zn), iron (Fe), barium (Ba), manganese (Mn) and aluminum (Al) in the red-boned goats were significantly higher than those of common goats (***P***<0.05). The level of silicon (Si) was significantly increased in the femoral diaphysis of the red-boned goats, and the copper (Cu) content was higher in the distal femur of the red-boned goats when compared with the common goats (***P***<0.05) (**Table 1**).

**Table 1:** The level of bone mineral composition of the femur diaphysis and the distal femur in red-boned (n = 3) and common (n = 3) Guishan goats (mg/kg).

	Femur diaphysis		Distal femur	
	Red-boned goats	Common goats	Red-boned goats	Common goats
Calcium (Ca)	270100±5478.5*	250400±5776.5	189400±4343.6*	170550±4899.2
Phosphorus (P)	136600±1133.36*	126950±1166.8	91175±577.1*	82240±578.7
Magnesium (Mg)	5402±388.4*	5236±355.2	3784.5±234.9*	3379.5±244.1
Sodium (Na)	7067±669.8	6953±667.6	5967.5±474.1	5861.5±488.5
Sulphur (S)	732±49	722±51.2	780±54.3	784.3±55
Barium (Ba)	106±11.3*	78.53±8.25	101.5±8.6*	86.5±6.9
Potassium(K)	347±33.4	338±35.1	453.5±41.8	451.8±42.3
Iron (Fe)	11.44±4.8*	5.13±1.5	18.8±4.35*	11.7±4.6
Copper (Cu)	1.77±0.16	1.7±0.21	2.55±0.43*	1.55±0.11
Silicon (Si)	29.9±4.8*	20.4±2.7	23.55±4.7	23.85±4.0
Zinc (Zn)	110±11.3*	87±9.8	88±7.6*	72±8.1
Manganese (Mn)	2.15±0.22*	1.76±0.17	1.95±0.32*	1.07±0.15
Aluminum (Al)	18.85±1.4*	9.15±1.1	33.5±3.5*	16.95±3.6

Data are expressed as the means ± SD;

* Significant difference between red-boned goats and common goats of the same age at ***P***<0.05, determined by Student's *t*-test.

### Assessment of mechanical properties

The left femoral diaphysis of each goat was used in a three-point bending test. In the three-point bending test, the bone elastic modulus (*Eb*) and yield stress (σ_yield_) of the red-boned goats was increased significantly when compared with those of the common goats (***P***<0.05), and the maximum energy (*U*) showed a significant difference between the red-boned and common goats (***P***<0.05). The ultimate stress (σ_ult_) was higher and the yield strain (ε_yield_) and ultimate strain (ε_ult_) were lower in the red-boned goats than in the common goats (***P***<0.05) (**Table 2**).

**Table 2:** Mean and SD values for mechanical properties obtained from three-point bending of the femur in the red-boned (n = 3) and common (n = 3) goats.

	*U* (J)	*Eb* (GPa)	σ_yield_ (MPa)	σ_ult_ (MPa)	ε_yield_ (%)	ε_ult_ (%)
Red-boned goats	10.88±0.77*	3561.14±410.3*	256.38±33.8*	358.30±37.9*	8.52±1.11	11.77±2.88
Common goats	9.03±1.00	3244.84±412.3	230.74±30.4	338.40±38.6	10.36±1.16*	14.02±2.89*

Data are expressed as the means ± SD;

* Significant difference between red-boned goats and common goats of the same age at ***P***<0.05, determined by Student's *t*-test.

### Microstructural changes in the distal femoral metaphysis and mid-femoral diaphysis

**Figure 3:**
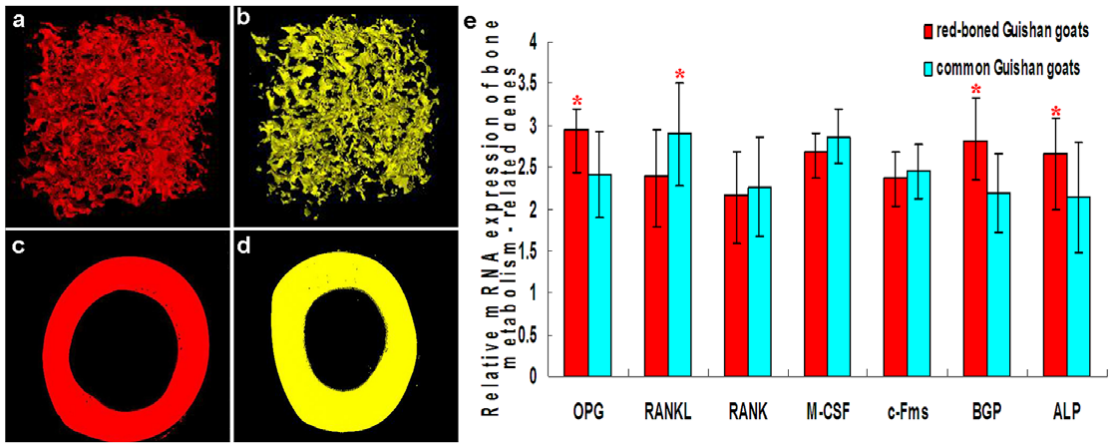
Three-dimensional images of microarchitectural properties in femur of goats. Three-dimensional images of the distal femur in red-boned goats (**a**) and common goats (**b**) obtained using micro-CT. Representative micro-CT images of the mid-femoral diaphysis in red-boned goats (**c**) and common goats (**d**). Relative mRNA expression of bone metabolism-related genes in the bone marrow of red-boned and common goats (**e**). Values are the means ± SD. *Significant difference between red-boned goats and common goats of the same age at ***P***<0.05, determined by paired-samples *t*-test.

Three-dimensional reconstruction of the trabecular bone structure in the distal femoral metaphysis by Micro-CT is shown in **Figure 3**. The distinct plate-like structure of the bone was displayed in the red-boned goats (**Figure 3a**), and the connecting rods were well maintained. In the common goats (**Figure 3b**), however, the plate-like structure was reduced, and many of the connecting rods were missing when compared with red-boned goats.

Compared with common goats, the red-boned goats showed a significant increase in the trabecular bone volume fraction (BV/TV), the trabecular thickness (Tb.Th), trabecular number (Tb.N), the connectivity density (Conn.D), and the bone mineral density (BMD). Correspondingly, a significantly lower trabecular separation (Tb.Sp) and structural model index (SMI) were observed in the distal femur of the red-boned goats (***P***<0.05) (**Table 3**).

**Table 3:** 3-D microstructural properties of the distal femoral metaphyseal trabecular bone in the red-boned (n = 3) and common (n = 3) goats at 18 months of age.

	BV/TV (%)	Tb.Th (mm)	Conn.D (/mm^3^)	Tb.N (/mm)	Tb.Sp (mm)	SMI	Distal femur BMD (g/cm^2^)
Red-boned goats	23.41±4.23*	0.241±0.08*	70.7±8.2*	4.03±0.11*	0.18±0.014	0.222±0.011	0.56±0.04*
Common goats	10.93±3.14	0.178±0.07	63.5±8.7	3.45±0.13	0.26±0.016*	0.345±0.013*	0.33±0.06

Data are expressed as the means ± SD;

* Significant difference between red-boned goats and common goats of the same age at ***P***<0.05, determined by Student's *t*-test.

The micro-structural differences in the mid-femoral diaphysis of the red-boned and common Guishan goats are shown in **Figures 3c, 3d**. The mean thickness, inner perimeter, outer perimeter, marrow area, cortical area, total area and mean BMD in the femoral diaphysis of the red-boned goats were significantly greater, by 22.61%, 26.03%, 8.60%, 53.31%, 15.38%, 18.14% and 46.23% (***P***<0.05), respectively, when compared with those of the common goats (**Table 4**).

**Table 4:** 3-D microstructural properties of the mid-femoral diaphyseal cortical bone in the red-boned (n = 3) and common (n = 3) goats at 18 months of age.

	Mean Thickness (mm)	Inner Perimeter (mm)	Outer Perimeter (mm)	Marrow Area (mm^2^)	Cortical Area (mm^2^)	Total Area (mm^2^)	Femur diaphysis BMD (g/cm^2^)
Red-boned goats	3.85±0.56*	36.02±3.56*	60.25±10.87*	102.46±16.98*	143.81±21.9*	246.27±32.1*	1.36±0.33*
Common goats	3.14±0.51	28.58±3.44	55.48±10.34	66.83±12.11	124.64±22.5	208.46±34.6	0.93±0.2

Data are expressed as the means ± SD;

* Significant difference between red-boned goats and common goats of the same age at ***P***<0.05, determined by Student's *t*-test.

### The mRNA expression of bone metabolism-related factors in the bone marrow

The mRNA expression of OPG, ALP and BGP in the red-boned goats was significantly higher (***P***<0.05) than that in common goats. The mRNA expression of M-CSF, RANK, and c-Fms in red-boned goats was lower than in common goats. There were no significant differences in M-CSF, RANK, and c-Fms mRNA expression between the red-boned and common goats. The mRNA expression of RANKL in common goats was significantly higher than that in red-boned goats (***P***<0.05) (**Figure 3e**).

### Rat experiments

**Figure 4:**
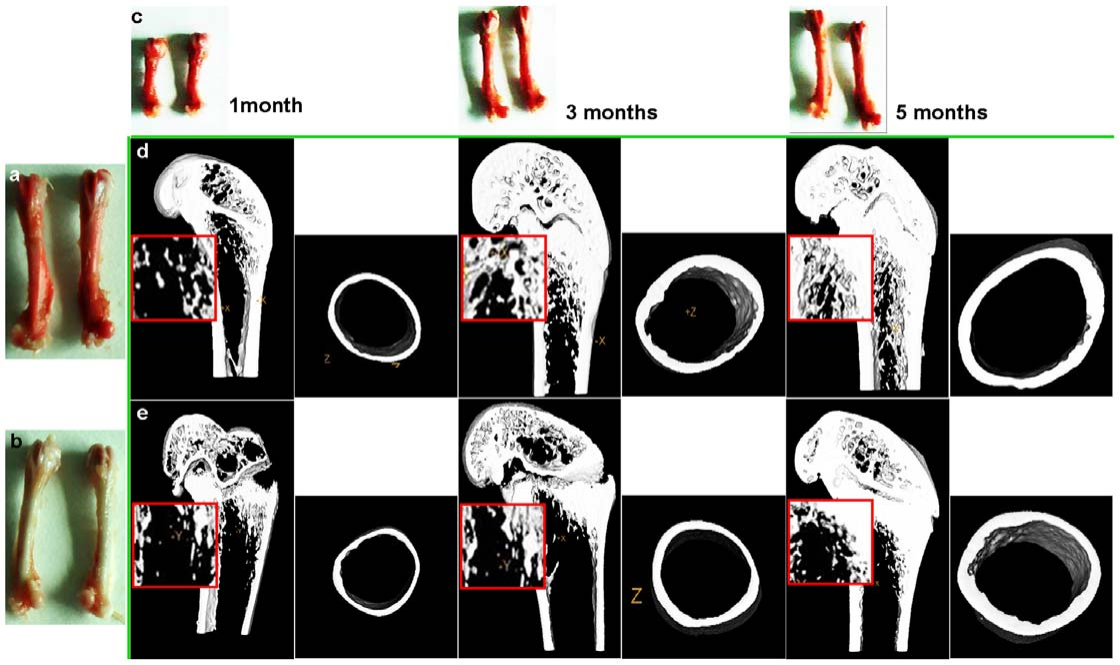
Three-dimensional images of microarchitectural properties in femur of rats. **a.** The femurs of rats from the T (test) group after 5 months of pseudopurpurin feeding. **b.** The femurs from the C (control) group rats after 5 months. **c.** The femurs of the T group rats at 1, 3 and 5 months after pseudopurpurin feeding. **d.** Depicts 3D and 2D (rectangle) images of the distal femur and mid-femoral diaphysis in T group rats after 1, 3 and 5 months of pseudopurpurin feeding. **e.** Depicts 3D and 2D (rectangle) images of the distal femur and mid-femur diaphysis in C group rats after 1, 3 and 5 months.

Certain bone tables of the rats were stained red on the periosteal and endosteal surfaces in the T group compared with C group after 1, 3 and 5 months of pseudopurpurin feeding (**Figure 4a, 4b, 4c**).

After 1 month of pseudopurpurin feeding, the mean thickness, inner perimeter, outer perimeter, marrow area, cortical area, total area and BMD of the femoral diaphysis, and the BV/TV, Tb.Th, Tb.N, Tb.Sp, SMI, Conn.D and BMD of the distal femur in the T group rats were not significantly from those of the C group rats (all ***P***>0.05).

After 3 months of pseudopurpurin feeding, the inner perimeter and marrow area of the femoral diaphysis, and the BV/TV, Tb.Th and Tb.N of the distal femur were significantly increased in the T group rats compared with those in the C group rats (all ***P***<0.05). The SMI and Tb.Sp were significantly decreased in the distal femurs of the T group rats (all ***P***<0.05)

After 5 months of pseudopurpurin feeding, the mean thickness, inner perimeter, outer perimeter, marrow area, cortical area, total area and BMD of the femoral diaphysis, and the BV/TV, Tb.Th, Tb.N, Conn.D and BMD of the distal femur in the T group rats were significantly greater than those in the C group rats (all ***P***<0.05). The SMI and Tb.Sp were significantly decreased in the distal femur in the T group rats (all ***P***<0.05) (**Figure 4d, 4e**) (**Table 5** **,** **Table 6**).

**Table 5:** 3-D microstructural properties of the mid-femoral diaphyseal cortical bone in test (T) and control (C) group rats after 1, 3 and 5 month of pseudopurpurin feeding (n = 10, in every batches).

	C group rats			T group rats		
	1 month	3 months	5 months	1 month	3 months	5 months
Mean Thickness (mm)	0.251±0.01	0.344±0.03^a^	0.385±0.01^a^	0.259±0.01b,c	0.371±0.02a,d	0.444±0.02a,b,c,d,e
Inner Perimeter (mm)	6.65±1.0	6.83±1.22	7.51±1.22a,b	6.73±0.88^c^	7.52±1.12a,b,d	8.49±1.34a,c,d,e
Outer Perimeter (mm)	8.34±1.54	10.22±1.34^a^	10.87±1.71a,b	8.41±1.33b,c	10.32±1.56a,c,d	13.95±1.87a,b,c,d,e
Marrow Area (mm^2^)	3.43±0.67	3.84±0.93^a^	4.04±0.94a,b	3.47±0.65a,b,c	4.23±0.91a,b,c,d	4.68±0.97a,b,c,d,e
Cortical Area (mm^2^)	1.90±0.36	3.26±1.00^a^	3.77±0.97a,b	1.95±0.34b,c	3.39±0.94a,c,d	4.54±0.92a,b,c,d,e
Total Area (mm^2^)	5.34±0.64	7.05±1.3^a^	7.12±1.31^a^	5.36±0.55b,c	7.08±1.4a,d	7.91±1.13a,b,c,d,e
Femur diaphysis						
BMD (mg/cc)	825.7±81.1	1098.1±88.2^a^	1158±99.5^a^	824.6±79.3b,c	1126.3±87.5a,d	1268.6±101a,b,c,d,e

Data are expressed as the means ± SD;

a,b,c,d,e ***P***<0.05, vs. 1 month (C groups), 3 months (C groups), 5 months (C groups), 1 month (T groups) and 3 months (T groups) after pseudopurpurin feeding respectively (Bonferroni correction test).

**Table 6:** 3-D micro-structural properties of the distal femoral metaphyseal trabecular bone in test (T) and control (C) group rats after 1, 3 and 5 month of pseudopurpurin feeding (n = 10, in every batches).

	C group rats			T group rats		
	1 month	3 months	5 months	1 month	3 months	5 months
BV/TV (%)	0.891±0.14	2.88±0.3^a^	6.29±1.5a,b	0.86±0.15b,c	4.37±1.5a,b,c,d	16.76±4.3a,b,c,d,e
Tb.Th (mm)	0.059±0.005	0.097±0.007a,b	0.13±0.01a,b	0.057±0.004b,c	0.128±0.01a,b,c	0.20±0.02a,b,c,d,e
Conn.D (/mm^3^)	17.47±1.33	31.55±6.5^a^	34.97±6.7^a^	17.9±1.28b,c	30.297±6.4a,d	43.60±8.5a,b,c,d,e
Tb.N (/mm)	2.56±0.71	3.07±0.89^a^	3.72±0.94a,b	2.66±0.78b,c	3.85±0.86a,b,d	4.19±0.94a,b,c,d,e
Tb.Sp (mm)	0.361±0.023	0.287±0.01	0.186±0.005a,b	0.347±0.03^c^	0.200±0.011a,b,d	0.127±0.005a,b,c,d,e
SMI	2.21±0.93	1.83±0.74^a^	1.12±0.08a,b	2.264±0.89b,c	1.42±0.76a,b,c,d	0.988±0.07a,b,c,d,e
Distal femur BMD (mg/cc)	160.1±33.8	246.7±57.4^a^	255.95±66.2^a^	159.87±34.2b,c	262.85±56.8a,d	322.51±65.2a,b,c,d,e

Data are expressed as the means ± SD;

a,b,c,d,e ***P***<0.05, vs. 1 month (C groups), 3 months (C groups), 5 months (C groups), 1 month (T groups) and 3 months (T groups) after pseudopurpurin feeding respectively (Bonferroni correction test).

In the three-point bending test, the elastic modulus (*Eb*), energy (*U*), yield stress (σ_yield_), ultimate stress (σ_ult_), yield strain(ε_yield_) and ultimate strain (ε_ult_) of the femoral diaphysis in the T and C groups tended to increase after 1, 3 and 5 months of pseudopurpurin feeding. The *Eb*, *U*, σ_yield_, σ_ult_, ε_yield_ and ε_ult_ of the femoral diaphysis in the T group rats were not significantly different from those of the C group rats after 1 and 3 months of pseudopurpurin feeding (***P***>0.05). However, the *Eb*, *U*, σ_yield_, σ_ult_, ε_yield_ and ε_ult_ of the femoral diaphysis in the T group rats were significantly different from those of the C group rats after 5 months of pseudopurpurin feeding (***P***<0.05) (**Table 7**).

**Table 7:** Mean and SD values for the mechanical properties obtained from the three-point bending of the femur diaphysis in the T and C groups of rats after 1, 3 and 5 month of pseudopurpurin feeding (n = 10, in every batches).

	C group rats			T group rats		
	1 month	3 months	5 months	1 month	3 months	5 months
*U* (mJ)	180.75±11.89	250.78±13.64^a^	323.08±19.7a,b	180.28±11.77b,c	248.19±14.3a,c,d	387.11±18.88a,b,c,d,e
*Eb* (MPa)	4949.18±110.3	8806.77±129.7^a^	13224.32±154.6a,b	5130.02±110.3b,c	8956.91±131.9a,c,d	16464.35±157.8a,b,c,d,e
σ_yield_ (MPa)	65.26±10	110.3±16.9^a^	125.60±22.8a,b	65.57±9.7b,c	112.14±15.4a,c,d	159.43±23.5a,b,c,d,e
σ_ult_ (MPa)	85.10±9.1	132.19±12.5^a^	156.24±23.5a,b	85.10±8.6b,c	136.62±11.1a,c,d	191.35±20.2a,b,c,d,e
ε_yield_ (%)	1.89±0.89	2.32±0.98^a^	3.27±1.34a,b	2.45±0.88b,c	3.17±0.78a,c,d	3.74±0.66a,b,c,d,e
ε_ult_ (%)	2.48±0.73	3.17±0.92^a^	3.94±1.6a,b	2.7±0.65b,c	3.99±0.85a,c,d	4.52±1.1a,b,c,d,e

Data are expressed as the means ± SD;

a,b,c,d,e ***P***<0.05, vs. 1 month (C groups), 3 months (C groups), 5 months (C groups), 1 month (T groups) and 3 months (T groups) after pseudopurpurin feeding respectively (Bonferroni correction test).

**Figure 5:**
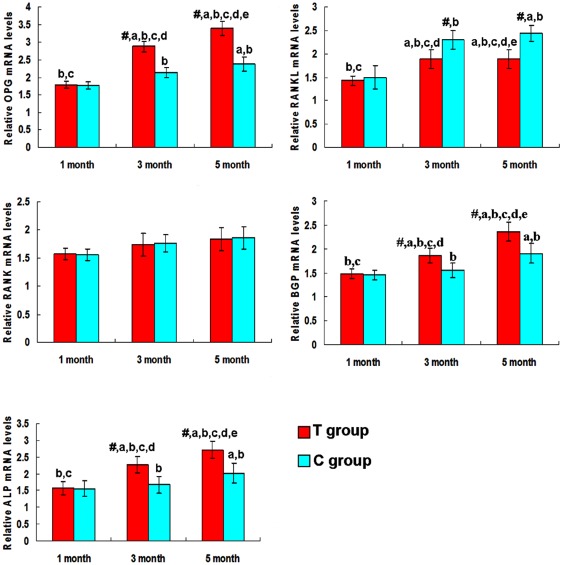
Relative mRNA expression of bone metabolism-related genes in the bone marrow of rats. Relative mRNA expression of osteoprotegerin, nuclear factor kappa B ligand, nuclear factor kappa B, osteocalcin and alkaline phosphatase in the bone marrow of the T and C group rats after 1, 3 and 5 months of pseudopurpurin feeding to the T group. The values are the means ± SD. **^#^** Significant difference between T group and C group rats of the same age at ***P***<0.05, determined by paired-samples ***t***-test. **^a,b,c,d,e^** ***P***<0.05, vs. 1 month (C group), 3 months (C group), 5 months (C group), 1 month (T group) and 3 months (T group) after pseudopurpurin feeding respectively (Bonferroni correction).

After normalization with GAPDH, the mRNA expression of OPG, RANKL, ALP and BGP was evaluated in the bone marrow samples obtained from rats in the T and C groups (**Figure 5**). The mRNA expression of ALP and BGP in the rats in the T group was significantly higher than that of those in the C group after 5 months of pseudopurpurin feeding (***P***<0.05). The ALP and BGP gene expression increased gradually over time. The OPG mRNA in the T group was markedly higher after 3 and 5 months of pseudopurpurin feeding. The RANKL mRNA in the T group had decreased significantly after 5 months of pseudopurpurin feeding compared with that in the C group (***P***<0.05).

## Discussion

Red-colored bones were found initially in some Guishan goats. However, the nature of the material that is responsible for the red color of the bone in the red-boned goats was unknown. Pseudopurpurin was identified by HPLC-ESI-MS and NMR analysis in the colored material that was extracted from the red bones of 18-month-old red-boned goats. This compound originates only from *Rubia cordifolia* L. (madder), which is a weedy perennial herbaceous plant. Its roots are the source of a natural dye [1]. Madder, which grows in the Guishan Mountains, can be eaten by goats, which subsist mainly by grazing. Consequently, their bones may be stained with pseudopurpurin, which is one of the main dyes in madder. It is unknown why some goats eat madder and others do not. Based on our knowledge, grazing goats have an opportunity to eat grass, and individuals have the choice to forage for grass. The phenomenon where by individual animals in a group choose to eat different diets has been described occasionally in wild animals, especially grazing animals. To confirm further the effect of pseudopurpurin on stained bone, an experiment was conducted that involved feeding Wistar rats for 5 months on diets to which 0.5% pseudopurpurin had been added. The bones of the rats that were fed pseudopurpurin displayed a red color, similar to that of the goats.

In red-boned goats, the levels of bone mineral composition in femurs stained with pseudopurpurin were increased significantly compared with those of common goats. To understand the events that occur during the deposition of pseudopurpurin and bone mineral, it may be of assistance to refer to the processes of calcification and ossification. A rather complicated structural formula for the calcium–aluminum–alizarin compound in bone was suggested by Rutishauser in 1940 [2]. Kiel and Heertjes [3]–[6] investigated the composition and structure of compounds formed by alizarin and its 3-derivatives with calcium, aluminum and various other metals. Pseudopurpurin resembles alizarin because it forms a colored metal salt that is highly insoluble in water [7]. Therefore, it is possible that an increased deposition of the principal salts in the red bone is associated with pseudopurpurin, which has a selective affinity for the principal salts of bone. Minerals formed in vitro have been found to consist of calcium and phosphorus deposited on well-banded collagen fibrils, and some of the crystals have matured into hydroxyapatite crystals [8], [9]. Zinc and magnesium are bound closely to apatite crystals, and zinc is essential for the formation of alkaline phosphatase in osteoblasts and for the synthesis of collagen and chondroitin sulfate [10]. Manganese exerts a beneficial effect on the skeleton and its degree of mineralization [11]. Therefore, a high level of bone mineral enhances bone BMD and leads to increased calcific deposits.

Low BMD is a major risk factor for fracture [12], but many investigators have also reported close correlations between microstructural properties and the biomechanical strength of bone [13]–[15]. The red-boned goats showed a significant alteration in bone geometry and architecture in the metaphyseal regions of the distal femur and at the midpoint of the femoral diaphysis when compared with common goats at 18 months of age. After 5 months of pseudopurpurin feeding, the bone microstructure of the distal femurs and femoral diaphyses of the rats were similar to those found in red-boned goats. The above results indicate that the increase in BMD and alterations in bone geometry and architecture found in red-boned goats are related to enhancement of the deposition of the principal salts in the red bone, which is associated with a selective affinity of pseudopurpurin for the principal salts of bone. The principal salts of the bone are distributed in a calcified matrix and come into contact with the osteoid tissue of developing bone.

Consistent with the evaluation of microstructural properties, in the bending test the elastic modulus, yield stress (σ_yield_) and ultimate stress (σ_ult_) of the bones from the red-boned goats were significantly increased at 18 months of age. After 5 months of pseudopurpurin feeding, the mechanical properties were also significantly enhanced in rats. The parameters of cortical bone structure and bone minerals are associated with the elastic modulus and ultimate load of the mid-femur [16]. Extensive research has shown that the mechanical properties of cortical and cancellous bone are closely related to the physical bone density [17], [18]. The BMD is associated with improvement in bone microarchitecture and biomechanical properties [19]. Therefore, we concluded that in the red-boned goats, the improved structure and mechanical properties of the bones result from the high concentrations of calcium salts in the bone. Pseudopurpurin–calcium compounds are held in solution by the colloids of blood plasma [7]. A colloidal solution of the calcium salt, added to bone gelatin, was much more stable on staining than pseudopurpurin itself, which would promote deposition of calcium compounds in bone tissue [20].

Calcium supplementation has been reported to result in an increase in bone formation through changes in the cytokine system, such as an increase in OPG concentration and decrease in RANKL concentration [21]. Importantly regulatory mechanisms in Ca^2+^ homeostasis are provided by OPG and RANKL [22]. In the current study, when compared with common goats, OPG mRNA expression was higher and RANKL mRNA expression was lower in red-boned goats. Indeed, in the red-boned goats, in the presence of low levels of M-CSF, RANKL appears to be not necessary or sufficient for the complete differentiation of osteoclast precursor cells into mature osteoclasts [23]. The decreased ratio of RANKL to OPG in the bones of red-boned goats contributes to the decreased differentiation and activation of the osteoclast. This interaction promotes osteoblast proliferation and differentiation and consequently prevents bone resorption. A higher level of ALP and BGP mRNA expression was observed in red-boned goats. The expression of ALP mRNA is currently regarded to be a major feature of the so-called osteoblastic phenotype, and it has long been suggested to play a specific role in mineralization [24]. Expression of BGP mRNA can lead to specific binding of hydroxyapatite, which promotes the deposition of bone salt to form hydroxyapatite crystals [25], [26]. In addition, the ratio of RANKL to OPG in the rats decreased, and the BGP and ALP mRNA expression levels were markedly upregulated, after 5 months of pseudopurpurin feeding. These results are consistent with those in the red-boned goats.

The red-colored material in the bones of red-boned goats is pseudopurpurin, which originates in the madder plant that is eaten by some goats during grazing. The increase in the BMD and the alterations in bone geometry and architecture observed in red-boned goats contribute to the enhanced deposition of the principal salts in the red bone. This may be associated with a selective affinity of pseudopurpurin for the principal salts of bone. The data obtained in this study should provide new insights into the improved bone metabolism, geometry and architecture induced by pseudopurpurin in humans and other mammals. The results provide a new scientific and theoretic basis for further study of the effects of pseudopurpurin on bone mineralization and bone microstructure.

## Materials and Methods

### Ethics statement

This study was carried out in strict accordance with the recommendations in the Guide for the Care and Use of Laboratory Animals of the National Institutes of Health. All animal studies were conducted according to the experimental practices and standards approved by the Animal Welfare and Research Ethics Committee at Jilin University (*Permit Number*: 20090719-1). All surgery was performed under sodium pentobarbital anesthesia, and all efforts were made to minimize suffering.

### Goats

Twelve goats were selected randomly, including common goats (n = 6, 18 months of age, body weight 30–35 kg) and red-boned goats (n = 6, 18 months of age, body weight 40–45 kg). All goats were grazed under conventional conditions with free access to water and diet in the same environment.

At necropsy, the red color was observed to be distributed throughout the whole skeleton of the red-boned goats. The whole left femur was used for dual energy X-ray absorptiometry (DEXA) studies. The left femoral diaphysis and the distal femur (n = 3, red-boned goats; n = 3, common goats) were used prior to submission for micro-CT analysis. Subsequently, the red material was analyzed. The left femoral diaphysis and the distal femur (n = 3, red-boned goats; n = 3, common goats) were evaluated using real-time PCR, a test of mechanical properties, and inductively coupled plasma mass spectrometry (ICP-MS).

### Extraction of the red material

The red material was isolated from dry bone powder (80°C) obtained from the red-boned goats using dilute hydrochloric acid (10%) and methyl benzene. After intense shaking at 37°C, the methyl benzene solution became pink. A red suspension was made using sodium hydrate solution and extracted repeatedly using hydrochloric acid. We removed the floating red suspension, heated it in a water bath and subjected it to reflux for 12 hours. This step was performed to clean the fat from the bone tissue. Subsequently, the red material that was obtained was centrifuged at 5000 *rpm* for 10 min. We transferred the floating red suspension to another sample tube, and the red suspension was dissolved in dimethyl sulfoxide.

### High-performance liquid chromatography–electrospray ionization–mass spectrometry (HPLC-ESI-MS)

A thermo ACCELA system (USA), equipped with an ACCELA PDA Detector, an ACCELA Auto sample and an ACCELA 1250 pump, was used to obtain chromatograms. Separation was performed on an ACUITY UPLC® BEH C 18 column (1.7 µm, 50 mm×3 mm, Waters, USA). Gradient elution was performed using a 20 mmol.L^−1^ ammonium acetate solution in water as mobile phase A and methanol as mobile phase B. The gradient composition was changed from 0 to 95% B over 25 min and then to 100% B over 5 min; the 100% value was held for 20 min. The flow rate was set to 1 mL min^−1^ and the injection volume was 20 µL. The UV detection was at 255 nm.

An LTQXL system (Thermo, USA) with an ESI ion source in the negative ion mode was used for a qualitative analysis of the compounds. The linear gradient conditions were the same as those used for UPLC-UV analysis. Elution was performed at a solvent flow rate of 0.4 mL/min, and a portion of the column effluent (0.2 mL/min) was delivered to the ion source for mass spectrometry. The conditions of the MS analysis were as follows: sheath gas flow rate: 40 arbitrary units; auxiliary gas flow rate: 5 arbitrary units; sweep gas flow rate: 1 arbitrary units; spray voltage (kV): 4; capillary temperature (°C): 250; capillary voltage (V): 22.0; and tube lens: 100.

### Nuclear magnetic resonance spectroscopy (NMR)

The ^1^H and ^13^C NMR analyses of the red material that was isolated from the red-colored bone from red-boned goats were performed on a Bruker 400 MHz nuclear magnetic resonance spectrometer (Faellanden, Switzerland) at 25°C. The ^1^H and ^13^C chemical shift values are reported in ppm relative to DMSO-***d_6_*** (δ_H_ 2.5, 3.3 ppm and δ_C_ 39.5 ppm).

### Bone mineral elements

For all of the goats, the samples of left femoral diaphysis and distal femur were cut first into pieces with a ceramic knife to avoid metal contamination, and the pieces were dried under a vacuum at 50°C to remove water and some volatile organic components. After pretreatment, samples were weighed precisely (0.5 g) into 30 mL vessels, and 5 ml of high purity HNO_3_ was added. The vessels were placed in a furnace at a temperature of 120°C until clean solutions were obtained. An inductively coupled plasma optical emission spectrometer (ICP-OES, iCAP6300, Thermo Scientific, USA) was employed for the analysis of the major elements, and an inductively coupled plasma mass spectrometer (ICP-MS, Xseries II, Thermo Scientific, USA) was employed for the analysis of the minor elements.

### Dual energy X-ray absorptiometry

The whole left distal femur and femoral diaphysis were assessed by Dual energy X-ray Absorptiometry (DEXA, MEDILINK OSTEOCORE3, France). Prior to testing, they were brought slowly to room temperature in a saline bath. Scanning was performed with each bone positioned on its caudal surface.

### Bone micro-computed tomography scanning

All of the specimens were scanned using a desktop micro-CT system (eXplore Locus SP, GE Healthcare, USA). For image acquisition, the specimens were machined into cylinders that were 20 mm in diameter. The specimens were scanned with a 14 µm isotropic voxel size using a large tube-14 µm-150 min-ss-microtomography scan protocol. The scan protocol consisted of rotation through 210° at a rotation step of 0.4°; the X-ray settings were standardized to 80 kV and 80 µA, and the exposure time was 2960 ms per frame. The scan time was approximately 150 min per sample. Three-dimensional (3-D) surface renderings were performed using Mic-view V 2.1.2 3-D reconstruction software. Regions of interest (ROIs) were reconstructed and analyzed by micro-CT with the same thresholds. The database was analyzed to give the 3-D parameters of each ROI. Each pair of these parameters in the same specimen was compared for each group.

**Figure 6:**
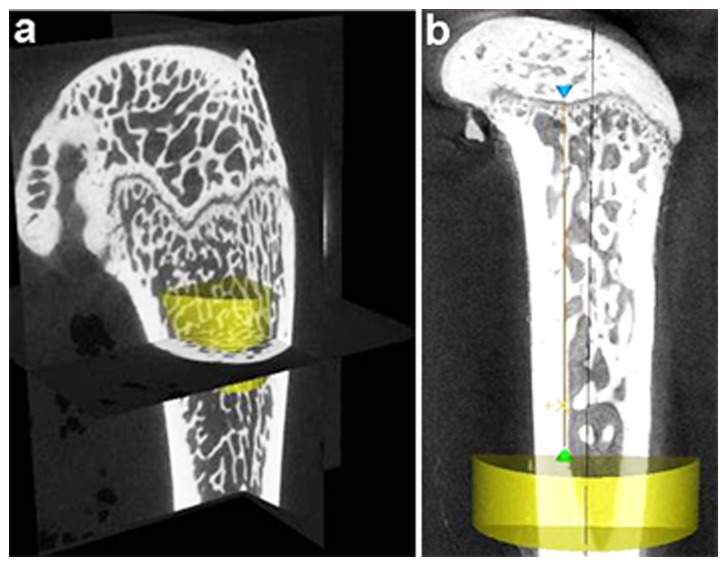
Region of interest for analysis of femur in goats and rats. **a.** Three-dimensional histomorphometric analysis was performed on the cancellous bone area 1 to 1.5 mm distal to the growth plate in the distal femur of goats and rats. **b.** The 2 mm thick cortical bone area in the mid-shaft of the fresh femoral diaphysis in rats and goats.

Three-dimensional morphometric analyses were performed in the 5×5×3 mm^3^ (goats) and 1.5×1.5×1.5 mm^3^ (rats) region of interest (ROI) on the area of cancellous bone 1 to 1.5 mm distal to the growth plate, using the distal femur (**Figure 6a**). The goat samples were scanned continuously with a thickness and increment of 20 µm for 120 slices. The rat samples were scanned continuously with a thickness and increment of 10 µm for 40 slices.

In addition to assessment of the cancellous bone of the distal femur, the cortical bone of the mid-shaft of the fresh femoral diaphysis was analyzed using the micro-CT method. Three-dimensional histomorphometric analysis was performed on a 2 mm thick ring of cortical bone from the mid-shaft of the fresh femoral diaphyses from the goats and rats (**Figure 6b**). The CT images were segmented into bone and marrow regions by applying the same, visually chosen, threshold for all of the samples.

The trabecular bone volume fraction (BV/TV) was calculated from the bone volume (BV) and total tissue volume (TV). Mean trabecular thickness (Tb.Th) was determined from the local thickness by the distance-transformation method. Trabecular separation (Tb.Sp) and trabecular number (Tb.N) were estimated on the basis of the plate model. The structural model index (SMI) is a parameter used to quantify the characteristic form of a three-dimensionally described structure in terms of the plate-like or rod-like nature of the complete structure. In addition, the connectivity density (Conn.D) of the trabecular bone was measured.

### Assessment of mechanical properties

The specimens were thawed to room temperature and hydrated prior to mechanical testing; otherwise, they were stored in saline-soaked gauze at −20°C for the duration of the study. The bones were thawed and kept fully moist before the mechanical testing. The left femoral diaphysis was tested with three-point bending using a materials-testing machine (MTS 858 System Inc., MN, USA). For three-point bending, the femoral diaphysis was placed with its anterior surface facing upward on two lower support bars 40 mm apart (goats) or 15 mm apart (rats at 1 month) and 20 mm apart (rats at 3 and 5 months), and a force in the opposite direction was applied to the midpoint. The holders were perpendicular to the horizontal axis, and the force was applied downward, perpendicular to the horizontal axis and at the midpoint of the specimen. A constant displacement rate of 2 mm/min was applied until destruction. Mechanical properties were measured, including maxiumum energy (*U*), the area of under the load–deformation curve, elastic modulus (*Eb*), the linear region of the load–deformation curve, yield stress (σ_yield_), the maximum point on the linear region of the curve, and ultimate stress (σ_ult_), the maximum point on the curve. Ultimate strain (ε_ult_) and yield strain (ε_yield_) were the strains measured when σ_ult_ and σ_yield_ were achieved [27].

### Real-time polymerase chain reaction analysis

Total RNA was extracted from the bone marrow of all of the goats and rats using Trizol (Invitrogen, USA), according to the manufacturer's instructions. The oligonucleotide primers and TaqMan probes for Osteoprotegerin (OPG), the receptor activator of the nuclear factor kappa B ligand (RANKL), the receptor activator of nuclear factor kappa B (RANK), macrophage colony stimulating factor (M-CSF), osteocalcin (BGP), alkaline phosphatase (ALP), and the macrophage colony stimulating factor receptor (c-Fms) high conservation sequence domain were used in the following technique.

OPG (goats), forward 5′-AGCG ACA CAA CTC ACG AGA AC-3′, reverse 5′- TTT CCA TCA ACT TTA GAA GCT GCT C-3′, Taqman Probe 5′- TGT CCA ATA TGC CTC CTC ACG CTG T-3′ (177-bp DNA fragment); RANKL (goats), forward 5′-TTC AGA ATT CCC CAG CCA GTA- 3′, reverse 5′- CCA AAA CCA GCA TCA AAA TCC -3′, Taqman Probe 5′- TGG TGC TTC CTC CTT TCA TCA GAG TGT G -3′ (80-bp DNA fragment); M-CSF (goats), forward 5′- CAG CAC AAG GAA GCC TCC AA -3′, reverse 5′- CCA GCA AGA CCA GGA TGA TAC TG -3′, Taqman Probe 5′- AGC TCC CTG GTT TTG TCT TCC GCC-3′ (82-bp DNA fragment); RANK (goats), forward 5′- GGG TTG CCA TGA ACT ATC AGT GA -3′, reverse 5′- AAC GAT CAA AGC AAC CAG TTT TTA -3′, Taqman Probe 5′- TGC AGC AAT GAA TGA CAC AGC CAG TTA -3′ (96-bp DNA fragment); c-Fms (goats), forward 5′- ATG GAG GTA GTG TGG ACG CC -3′, reverse 5′-CGA TCA CGT AGG CTT CCA AGA -3′, Taqman Probe 5′- TGG CCA CGC AGG TGT AGT TGC C -3′ (78-bp DNA fragment); BGP (goats), forward 5′- AGC GAG GTG GTG AAG AGA CT -3′, reverse 5′- CCT GGA AGC CGA TGT GGT-3′, Taqman Probe 5′- CAG ATC CGC TGG AGC CCA AGA GGG -3′ (145-bp DNA fragment); ALP(goats), forward 5′- GCA CCG CCA CCG CCT ACT TGT -3′, reverse 5′- GGT CAC GAT GCC CAC GGA TTT C -3′, Taqman Probe 5′- GGC CAA TGA GGG CAC GGT GGG GGT G -3′ (145-bp DNA fragment); and GAPDH (goats), forward 5′-GGC TGG GGC TCA CTT GAA G -3′, reverse 5′- TTC ACG CCC ATC ACA AAC ATG -3′, Taqman Probe 5′- TCA TCT CTG CAC CTT CTG CTG ATG C -3′ (87-bp DNA fragment).

OPG (rats), forward 5′-AGC GGC TGC CGC CTG AGG TT -3′ and reverse 5′-GTG AAA CAG GAG TGC AAC CG-3′, Taqman probe 5′-CCA GAA ACC GGA CGT CAG CTC TTG TGT GAC-3′ (127-bp DNA fragment); RANK (rats), forward 5′-ACT GGA AAC AGT AAC TCC AC-3′ and reverse 5′-TCC TCC TGC ACA GGG CGG CC-3′, Taqman probe 5′- CGC AGG AGG GCC CGG GTT CCG CAG AGC CC-3′ (152-bp DNA fragment); RANKL (rats), forward 5′-CTC CTG GGG CTG GGA CTG GG-3′ and reverse 5′-TAA AGT CAC TCT GTC CTC TT-3′, Taqman probe 5′-AAA ACG CAG ATT TGC AGG ACT CGA CTC TGG-3′ (90-bp DNA fragment); BGP (rats), forward 5′-AGT CCC ACA CAG CAA CTC GG-3′ and reverse 5′-TGT GTG AGC TCA ACC CCA AT-3′, Taqman probe 5′-ACA AAG CCT TCA TGT CCA AGC AGG AGG GCA-3′ (135-bp DNA fragment); and ALP (rats), forward 5′-GCACCGCCACCGCCTACTTGT-3′ and reverse 5′-GGT CAC GAT GCC CAC GGA TTT C -3′, Taqman probe 5′-GGC CAA TGA GGG CAC GGT GGG GGT G-3′ (125-bp DNA fragment). GAPDH (rats) was used as a reference gene as follows: forward 5′-AAA TGG TGA AGG TCG GTG TG-3′ and reverse 5′-TGA AGG GGT CGT TGA TGG-3′, Taqman probe 5′-TCA TCT CTG CAC CTT CTG CTG ATG C-3′ (108-bp DNA fragment).

Quantitative reverse-transcription polymerase chain reaction (qPCR) assays were performed on an ABI prism 7000 real-time PCR system (Applied Biosystems) with cycling conditions as follows: 5 min of denaturation at 95°C followed by 40 cycles of 95°C for 30 s and 60°C for 1 min (data collection). To correct the input RNA concentration, the relative gene copies were normalized further to the measurement for GAPDH.

### Rat experiment

The Wistar rats in the test group (T group, 1 month of age, n = 30) and control group (C group, 1 month of age, n = 30) were fed a commercially available pelleted diet as prescribed by the manufacturer to fulfill all of their dietary needs. The T group received an additional 0.5% of pseudopurpurin powder by weight in their diet; the C group did not receive this added constituent. The rats in each group were killed in batches at 1 (n = 10), 3 (n = 10), and 5 (n = 10) months after pseudopurpurin feeding by an overdose of sodium pentobarbital administered intraperitoneally. The micro-CT, assessment of mechanical properties and bone mineral density, and real-time PCR were performed as explained above.

### Statistical analyses

All data are presented as mean values ± standard deviation (SD.). Significance was defined as **P**<0.05. All statistical analyses were performed using the statistical package SPSS for Windows version 13.0.

In the goat experiments, the statistical analysis of the differences between red-boned goats and common goats at 18 months of age was performed with a Student's *t*-test.

In the rat experiments, the statistical analysis of the differences between the test (T) group and the control (C) group, at the same age, was performed with a one-way analysis of variance (ANOVA). This was used to assess the effect of group (test and control group rats) and time (1, 3 and 5 months after pseudopurpurin feeding). The post-hoc test was corrected for multiple comparisons by performing a Bonferroni correction test.
